# Application of trans-thoracic echocardiography and coronary CT angiography to diagnose mitral valve prolapse

**DOI:** 10.1186/s13019-019-0925-8

**Published:** 2019-06-24

**Authors:** Jingbo Li, Yun Zhang, Liulong Zhang, Deyong Lv, Hui Song, Haifeng Zhang, Guangyan Zhou, Hong Li

**Affiliations:** 1 Department of Ultrasound, Dongying People’s Hospital of Shandong, Dongying, 257091 China; 2 Department of Radiology, Dongying People’s Hospital of Shandong, No. 317, Nan Yi Road, Dongying, 257091 Shandong China; 3 Department of Ultrasound, Anorectal Disease Hospital, Victory Petroleum Administration Bureau, Dongying, 257077 China

**Keywords:** Trans-thoracic echocardiography, Coronary CT angiography, Mitral valve prolapse

## Abstract

**Objective:**

At present, multi-detector cardiac CT has been widely applied in the detection of heart valve morphology and function. This study aims to compare the coronary CT angiography and trans-thoracic echocardiography for patients with mitral valve prolapse.

**Methods:**

CT angiography and trans-thoracic echocardiography were adopted to detect the movement range and thickness of valvula bicuspidalis. The ultrasonic parameters of CT angiography were considered as standard reference value.

**Results:**

Receiver operating characteristic (ROC) curve showed that the area of mitral valve motion amplitude under ROC curve was 95% detected by CT angiography with statistical significance (*P* < 0.001). Based on the intercept point on the ROC curve, the sensitivity and accuracy of mitral valve amplitude detected by CT angiography was 69.2 and 95.6%, the false positive and false negative rate was 5.2 and 32.4%, the predicted value of positive and negative was 92.9 and 76.9% and the consistency rate of motion range and leaf thickness detected by CT angiography was 83.3 and 47.5%. Trans-thoracic echocardiography showed that the thickness and motion range of mitral leaflet was (1.95 ± 0.22) mm and (1.5 ± 2.28) mm. The thickness and motion range of mitral leaflet that detected by CT was (2.00 ± 0.54) mm and (3.76 ± 2.1) mm.

**Conclusion:**

The accuracy and reliability of CT angiography for patients with mitral valve prolapse are higher than those of trans-thoracic echocardiography.

## Introduction

Mitral valve prolapse is one of the most common heart valve diseases. Epidemiologic studies have demonstrated that the morbidity rate of mitral valve prolapse is approximately 3–6%. A majority of patients have no obvious clinical manifestations. If severe mitral regurgitation occurs in the late period, the patients have to undergo mitral valve repair. Therefore, the monitoring and accurate diagnosis of high-risk patients is of great significance [1–4]. If patients with moderate or severe mitral valve prolapse were complicated with the left ventricle and decreased contractile function, the incidence rate of cardiac death events will be increased significantly. We should monitor these patients closely. The clicking sound and / or noise can be heard during the middle or late stage of systole of mitral valve prolapse. At present, trans-thoracic echocardiography has been widely regarded as a standard method of diagnosing heart valve diseases. During the detection, the displacement of mitral valve leaflet is more than 2 mm or the long axis direction, which tends to the atrium cordis [5–8]. Nevertheless, trans-thoracic echocardiography also has some limitations that it depends on the experience of operators, body size and heart rate of patients, and related detection device, etc [9] Multi-detector CT could diagnose abnormal diseases and functions of cardiac valves effectively, and assess coronary artery disease. The application of this method in the evaluation of mitral valve prolapse has been rarely reported [10–12]. The aim of this study is to compare and evaluate these two diagnostic techniques, which could provide guidance and reference for clinicians.

## Materials and methods

### Baseline data

From August 1st, 2014 to July 31st, 2015, patients with mitral valve prolapse by physical examination and trans-thoracic echocardiography were recruited. Then, valve repair surgery was carried out and re-examined by CT scan at 3 months after initial diagnosis. Exclusion criteria: patients with renal insufficiency (serum creatinine> 1.5 mg/dl), thyroid diseases, iodine allergy, progressive heart failure, atrial fibrillation and other types of arhythmia, pregnant women, those with previous history of myocardial infarction, heart ischemia or other diseases occurring in recent 3 months and those unwilling to participate in this study were excluded. The diagnosis of mitral valve prolapse was confirmed by two experienced physicians, who reviewed the results of trans-thoracic echocardiography. If the heart rate was > 70 times/min before the examination, β-receptor blocking pharmacon (25–50 mg metoprolol) was injected to control the heart rate at 65 times/min. A total of 90 eiligible patients completed the study. Written informed consents were obtained from all patients or family members.

### Trans-thoracic echocardiography and data acquisition

Conventional trans-thoracic echocardiography was performed by experienced physician (GE Vingmed Ultrasound, VIVID-3, General Electrics, USA). Mitral valve prolapse was defined as valve cusp displacement > 2 mm or the parasternal direction of the long axis tended to the left atrial direction. Mitral valve leaflet thickness was measured under two- to four-chamber view similar to CT scan. The severe degree of mitral regurgitation, end-diastole and end-systole of left ventricular volume, ejection fraction, peak pulmonary arterial pressure and other parameters were also measured.

### CT angiography

Patients were processed by 64-row CT inspection after 3 months of trans-thoracic echocardiography (GE. Light speed. VCT, General Electrics, USA). The related parameters were set as followed: level alignment was 2 × 32 × 0.6 mm; profile acquisition was 64 × 0.6 mm; pitch of screw thread was 0.2; slew time was 350 ms; tube voltage was 120 kV; tube current time was 600 mAs. A 70-100 ml nonionic iodine contrast agent was applied in the detection, washed by 40 ml physiological saline and administered via intravenous dripping at a rate of 3 ml/ seconds along the elbow vein. The bolus technique was utilized to calculate the scanning delay.

### CT data acquisition

Besides the function of coronary artery with CT examination, the thickness and motion range of mitral valve leaflet were collected, which was 25–30% data during R-R period, because the motion range of valve cusp in this section reached the maximal value. The data was processed by the late stage of multi-planar reconstruction, we defined three-chamber plane generated in the direction of parasternal longitudinal axis wasdefined as passing through the left ventricular long axis and left ventricular outflow tract, equivalent to the left coronal oblique angle. Two-chamber plane was defined as the left ventricular geometric center vertical long axis direction, equivalent to the sagittal oblique view passing through the ventriculus sinister, left atrium and center of mitral valve. Four-chamber plane was generated by the recombining of left ventricular short axis, which was located in the middle of the left ventricle. It was defined as a transitional plane passing through the left ventricular center, diaphragm and right ventricular free wall. During the systole period (25–30% R-R period), the maximum vertical distance was recorded between the mitral valve leaflet and annulus plane under three-chamber view, equivalent to the long axis view of trans-thoracic echocardiography. Left ventricular offset was defined as negative value, and its left atrial offset was defined as positive value. The thickness of valve cusp was measured under two- to four-chamber view, excluding the calcified part. The explanation of checking image was finished by two experienced clinicians, who were blind to the results of trans-thoracic echocardiography.

### Statistical analysis

SPSS 20.0 statistical software was utilized for data analysis. The data were expressed as mean ± standard deviation (SD). Continuous variables were statistically compared by t-test. Classified variables were compared by chi-square test or Fisher exact test. The correlation analysis was processed by Pearson’s correlation analysis examination. The ROC curve was adopted to detect the cut-off point. The sensitivity, specificity, false positive, false negative and other parameters were detected and measured. A *P* value of less than 0.05 was considered as statistical significance.

## Results

A total of 90 patients were included in the study, aged (61.2 ± 13.4) years old on average, the male/female ratio was 3:2, (56.3 ± 11.9) years old for the male and (66.4 ± 12.7) years old for the female. The average systolic pressure, diastolic pressure and heart rate before trans-thoracic echocardiography and CT detection were (131 ± 12) mmHg vs. (128 ± 14) mmHg, (70 ± 8) mmHg vs. (72 ± 8) mmHg, (69 ± 10) times/min vs. (68 ± 9) times/min, respectively, as illustrated in Table 1.

**Table 1:** Baseline data of enrolled patients

Parameter (*n* = 90)	
Age (years)	61.2 ± 13.4
Male (%)	54 (60%)
BMI (kg/m^2^)	26.6 ± 3.8
No clinical symptom	28 (31.1%)
Had clinical symptom	62 (68.9%)
Related risk factors	
Diabetes mellitus (%)	9 (10%)
High blood pressure (%)	32 (35.6%)
Smoker (%)	40 (44.4%)
Dyslipidemia (%)	22 (24.4%)
Family medical history (%)	25 (27.8%)

### Trans-thoracic echocardiography

Trans-thoracic echocardiography showed that the thickness and motion range of mitral leaflet were (1.95 ± 0.22) mm and (1.5 ± 2.28) mm. The cardiac examination results were regarded as the reference standard. The thickness and motion range of mitral leaflet that detected by CT were (2 ± 0.54) mm and (3.76 ± 2.1) mm.

### CT angiography

ROC analysis of the data obtained by CT angiography showed that compared with the results of trans-thoracic echocardiography, the area under standard curve of valve leaf thickness detected by CT was 57% (95%CI: 0.42–0.76) with no statistical significance (*p* = 0.36). The optimal cut-off point of using CT to detect leaf thickness was 1.6 mm. The sensitivity of mitral valve prolapse was 94.2%, the specificity was 95.1%, the false positive was 5.1%, the false negative was 94.9%, the positive predictive value (PPV) was 48% and the negative predictive value (NPV) was 52%. The consistency between CT angiography and trans-thoracic echocardiography was 47.5%.

**Fig. 1:**
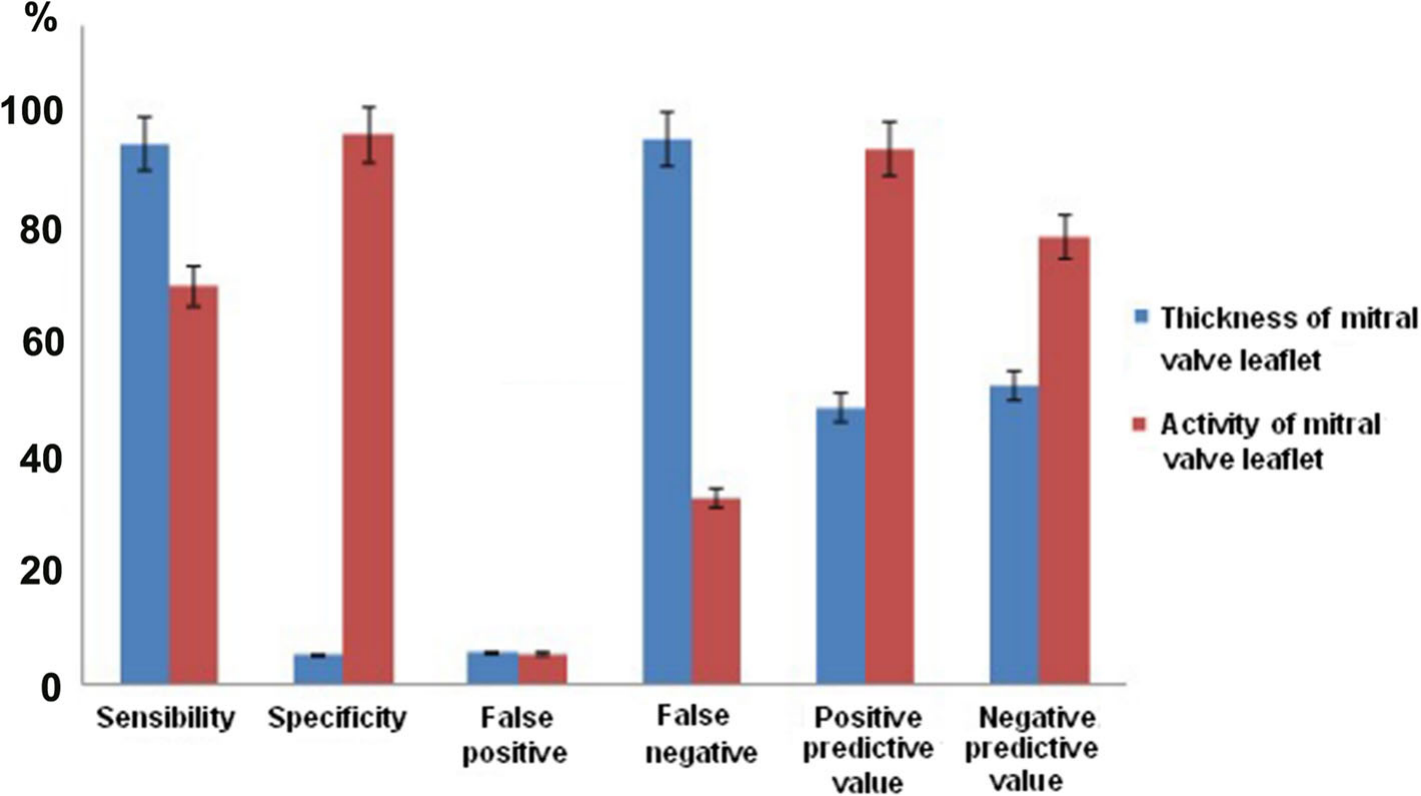
Relevant data percentage of mitral valve prolapse judged by mitral valve thickness and activity in CT detection

**Fig. 2:**
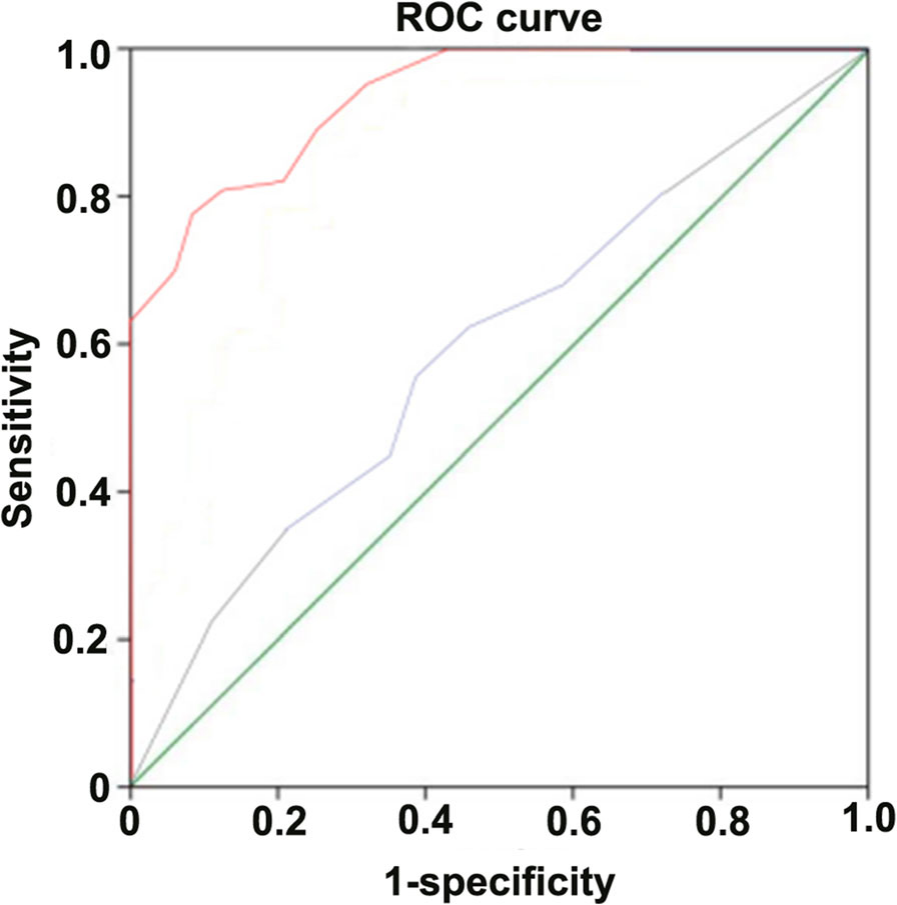
The area of mitral valve thickness and motion range under ROC curve detected by CT

CT angiography showed that the area of mitral valve leaflet movement amplitude under ROC curve was 95% (95%CI: 0.88–1.0) with statistical significance (*P* < 0.05). The motion range of valve cusp was a vital index of diagnosing mitral valve prolapse. The cut-off point in ROC curve was 2.5 mm, the sensitivity of mitral valve prolapse was 69.2%, specificity was 95.6%, the false positive was 5.2%, the false negative was 32.4%, the positive predictive value (PPV) was 93.1% and the negative predictive value (NPV) was 77.8%. The consistency between CT angiography and trans-thoracic echocardiography was 83.3%. (Fig. 1 and 2).

### Correlation analysis

According to the obtained data, 70% direct correlation existed between CT angiography and trans-thoracic echocardiography in the measurement of leaf thickness with statistical significance. The relativity of valve cusp motion range was 95%, which also had statistical significance (*P* < 0.05).

## Discussion

At present, CT detection has been used widely in clinical application, which has been utilized evaluate the coronary artery occlusion in the early stage and can improve the long-term clinical prognosis of patients. However, there is still a lack of study on mitral valve related disorders, such as the diagnostic value of mitral valve prolapse [13–16]. Before the mitral valve surgery, most patients require cardiac ultrasound examination to quantify the degree of mitral regurgitation and its anatomical characteristics. For a part of patients, they have to receive another coronary CT angiography to ensure whether the extra treatment should be delivered besides repairing valvula bicuspidalis [17–20]. Consequently, CT scan is relatively comprehensive and convenient. The aim of this study was to explore the differences between multi-slice spiral CT and trans-thoracic echocardiography in the diagnosis of mitral valve prolapse.

First, we analyzed the linear correlation between the thickness of valve cusp and advocacy motion range by Pearson’s correlation analysis and the results showed significant correlation between these two methods. Moreover, we also applied statistical cross-analysis for assessing the values between CT angiography and trans-thoracic echocardiography. The results showed that CT angiography possessed a higher specificity for diagnosing mitral valve prolapse and an acceptable sensitivity, which was basically the same with the results of existing related literature reports. In this study, the sensitivity of using CT angiography to diagnose mitral valve prolapse was 69.2%, identical to the sensitivity in Shah and other researchers’ reports. However, 67 patients were included in the study by Ghosh et al and the sensitivity of mitral valve prolapse detected by CT was 92%. Feuchtner et al. have demonstrated that the sensitivity in 112 patients is calculated as 96%. Therefore, with the increase of sample size, the sensitivity shows a rising trend because the sample size included in this study was relatively limited, the obtained sensitivity was relatively reasonable. The selection for diabetes mellitus, high blood pressure, mammary cancer and other diseases with high morbidity and death rate always required the detection methods with high sensitivity, while it was not needed to select for mitral valve prolapse, but the related study on large sample was still significant.

In this study, the specificity of mitral valve prolapse detected by CT angiography was 95.6%, the specificity in Shah, Ghosh, Feuchtner and other researchers’ investigations was 100, 97.1 and 93%, respectively. Consequently, CT was a relatively reliable method for diagnosing mitral valve prolapse, and the related false positive rate was low. The PPV of this detection method was higher (93.1%), but when applied CT angiography for detecting mitral valve leaflet movement (> 2.5 mm in this study), the diagnosis of mitral valve prolapse was established. The PPV in previous study was 87, 80 and 93%. The NPV in this study was 77.8%, which was basically the same with the result of 80, 83 and 91% in previous study. In the detection of mitral valve thickness, the relativity of these two methods was 70%, which indicated that the reliability of diagnosing mitral valve thickness was pretty well. The overall accuracy of mitral valve prolapse detected by CT angiography was 83.3%, so it was a reliable detection method.

## Conclusions

CT also has the advantage of less radiation exposure, side effects of contrast agent and lower cost, etc [20–24] The results of this study show that the accuracy and reliability of CT angiography for patients with mitral valve prolapse are higher than those of trans-thoracic echocardiography. CT angiography is a reliable method to deliver a diagnosis of mitral valve prolapse.

### Study limitation

This is a retrospective investigation. Therefore, the preliminary findings obtained in current article remain to be further validated in a prospective study. In addition, the detection methods can be widened and the sample size can be enlarged to confirm the obtained conclusion.

## Data Availability

The datasets used and/or analysed during the current study are available from the corresponding author on reasonable request.

## Abbreviations

NPV: negative predictive value
PPV: positive predictive value
SD: standard deviation
